# The REstart or STop Antithrombotics Randomised Trial (RESTART) after stroke due to intracerebral haemorrhage: statistical analysis plan for a randomised controlled trial

**DOI:** 10.1186/s13063-019-3270-2

**Published:** 2019-03-25

**Authors:** Rustam Al-Shahi Salman, Gordon D. Murray, Martin S. Dennis, David E. Newby, Peter A. G. Sandercock, Nikola Sprigg, Cathie L. M. Sudlow, David J. Werring, Philip M. White, William N. Whiteley

**Affiliations:** 1 Centre for Clinical Brain Sciences, University of Edinburgh, Chancellor’s Building, 49 Little France Crescent, Edinburgh, EH16 4SB UK; 2 Usher Institute of Population Health Sciences and Informatics, University of Edinburgh, Edinburgh, UK; 3 Centre for Cardiovascular Science, University of Edinburgh, Edinburgh, UK; 4 Faculty of Medicine & Health Sciences, University of Nottingham, Nottingham, UK; 5 Institute of Neurology, University College London, London, UK; 6 Institute of Neuroscience and Newcastle University Institute for Ageing, Newcastle-upon-Tyne, UK

**Keywords:** Secondary prevention, antiplatelet therapy, stroke, intracerebral haemorrhage, randomised controlled trial, statistical analysis plan

## Abstract

**Background:**

For adults surviving stroke due to spontaneous (non-traumatic) intracerebral haemorrhage (ICH) who had taken an antithrombotic (i.e. anticoagulant or antiplatelet) drug for the prevention of vaso-occlusive disease before the ICH, it is unclear whether starting antiplatelet therapy modifies the risks of recurrent ICH, major haemorrhagic events, major occlusive vascular events, or a composite of all major vascular events compared to avoiding antiplatelet therapy.

**Methods/design:**

The REstart or STop Antithrombotics Randomised Trial (RESTART) is an investigator-led, parallel group, open, assessor-blind, randomised trial comparing starting versus avoiding antiplatelet therapy for adults surviving antithrombotic-associated ICH. Recruitment began on 22 May 2013 and ended on 31 May 2018. Follow-up ended on 30 November 2018. This update to the protocol describes the statistical analysis plan (version 1.7, finalised on 25 January 2019). Database lock and un-blinding occurred on 29 January 2019, after which the un-masked trial statistician conducted the final analyses according to this statistical analysis plan.

**Discussion:**

Final results of RESTART will be analysed and disseminated in May 2019.

**Trial registration:**

ISRCTN registry 71907627. Prospectively registered on 25 April 2013.

**Electronic supplementary material:**

The online version of this article (10.1186/s13063-019-3270-2) contains supplementary material, which is available to authorized users.

## Background

Patients with stroke due to spontaneous intracerebral haemorrhage (ICH) often have past histories of vascular disease and people who survive ICH are at risk of vaso-occlusive events [1]. When patients develop ICH whilst taking antithrombotic (antiplatelet or anticoagulant) drugs for the prevention of vaso-occlusive disease, these drugs are usually immediately discontinued because of the risk of ICH growth in the 24 h after symptom onset [2–4]. If these patients survive the early consequences and complications of the ICH, secondary prevention of vaso-occlusive disease is an important consideration for many survivors [1, 3–6]. Blood pressure lowering is effective for the prevention of stroke after ICH [7, 8].

However, it is unknown whether survivors of ICH that occurred whilst taking antithrombotic drugs should start or avoid antiplatelet drugs for continued secondary prevention. A recent systematic review and meta-analysis of observational studies of all sub-types of intracranial haemorrhage found lower risks of ischaemic events and no difference in haemorrhagic events associated with antiplatelet drug resumption [9]. Small, non-randomised observational studies restricted to patients with stroke due to ICH have reported similar associations [10–14], but these associations were not ‘dramatic’ so randomised controlled trials (RCTs) are needed to resolve this therapeutic dilemma [15]. However, such RCTs have not been published [16]. Therefore, we designed the REstart or STop Antithrombotics Randomised Trial (RESTART) to determine whether antiplatelet drugs increase the risk of recurrent symptomatic ICH to an extent that might outweigh any beneficial reduction in vaso-occlusive disease [17].

## Methods/Design

### Trial design

The primary objective of this parallel group, open, assessor-blind, randomised trial is to estimate the relative and absolute effects of a policy of starting antiplatelet drugs versus avoiding antiplatelet drugs on the risk of recurrent symptomatic ICH after spontaneous ICH. We intended to recruit 720 participants (based on the sample size calculation described in the protocol [17]) and allocate them 1:1 to each treatment group using a minimisation algorithm based on five variables collected beforehand, namely (1) qualifying ICH location (lobar versus non-lobar, based on local investigator’s interpretation of scan); (2) time since ICH symptom onset (1–6 days, 7–30 days, > 30 days); (3) antiplatelet drug(s) that the patient’s physician would start if allocated (aspirin alone versus other antiplatelet regimen, including combination treatment); (4) participant age at randomisation (< 70 years vs. 70 years or older); and (5) predicted 6-month outcome (predicted probability of good outcome < 0.15 vs. ≥ 0.15 [18]). Since the primary objective of the trial is to estimate a treatment effect, the trial does not fit into a conventional superiority, equivalence or non-inferiority hypothesis testing framework.

RESTART collected all participants’ diagnostic brain imaging (usually computed tomography (CT), but sometimes magnetic resonance imaging (MRI) alone) that diagnosed ICH before randomisation. An imaging sub-study was also conducted involving brain MRI performed according to a specific imaging protocol to test for an interaction between imaging biomarkers of cerebral small vessel disease and the effects of antiplatelet drugs. The RESTART imaging panel collected radiographic data from these imaging studies, masked to treatment allocation group [17]. The analysis of the imaging sub-studies will be largely exploratory. The MRI sub-study will test hypotheses about microbleeds and explore hypotheses about other modifiers of the effect of antiplatelet drugs. The diagnostic CT imaging study will explore hypotheses about modifiers of the effect of antiplatelet drugs.

### Statistical interim analyses and stopping guidance

The final Data Monitoring Committee charter (version 1.6, 28 March 2014) did not specify a formal fixed schedule of interim analyses, but allowed the Data Monitoring Committee to advise the chair of the Trial Steering Committee and the sponsor if there was ‘proof beyond reasonable doubt’ that might also reasonably be expected to influence clinical practice. Application of this ‘overwhelming evidence’ principle makes adjustment of the final level of statistical significance testing unnecessary [19–21].

### Trial population

We did not ask collaborators at participating sites to record every patient they screened for eligibility, although some sites’ standard practice was to do this anyway, which enabled us to investigate reasons for non-recruitment of eligible patients at these sites [22]. We asked collaborators to report and record every patient for whom written informed consent was obtained, so we will report the numbers of patients with consent who were either randomised or not (with reasons). Participants found to be ineligible after randomisation will be quantified and categorised according to the eligibility criteria in the protocol [17], but they will be analysed in the treatment group to which they were allocated. Because RESTART uses multiple overlapping sources of ascertainment of outcomes during follow-up [17], we will only withdraw participants from analyses if the participant or their representative requests complete withdrawal from all methods of follow-up after randomisation. We will analyse data collected on the participant up to the point of withdrawal. We will not regard participants who change their allocated treatment as premature withdrawals. We will quantify adherence to the allocated treatment at hospital/clinic discharge after randomisation, and at the time of each subsequent follow-up postal/telephone questionnaire assessment (first done at 6 or 12 months after randomisation and then annually for up to 5 years). We will describe loss to follow-up in two ways. Firstly, we will quantify the proportion of patients without a complete follow-up questionnaire at each planned follow-up time point (first starting 6–12 months after randomisation). Secondly, we will quantify the sum of the total observed person-time for each participant in the trial as a proportion of the sum of their total potential person-time until the last planned trial follow-up questionnaire for that participant [23].

### Analysis populations

All analyses will follow the ‘intention-to-treat’ principle in order to fully preserve the benefit of randomisation. The intention-to-treat population will comprise all participants who have been randomised in RESTART, regardless of whether they were subsequently deemed ineligible after independent review of their diagnostic brain imaging. We will include all randomised participants in the analysis (irrespective of whether they adhere to the allocated treatment), all retained in the group to which they were allocated (i.e. ‘as-randomised’). However, in the brain imaging studies within RESTART, imaging may (1) not have proceeded despite consent being obtained; (2) not have been provided; (3) have been undertaken but may have contravened the required protocol; (4) have been undertaken, but was degraded by motion artefact; or (5) have demonstrated that the patient was ineligible for inclusion in RESTART (which precluded collection of ratings by the RESTART Imaging Panel). Therefore, patients will be included in the CT imaging study and the MRI sub-study datasets if their pre-randomisation brain imaging was readable and confirmed their eligibility for RESTART.

### Baseline characteristics

We will summarise the following baseline characteristics overall and by treatment group:

- The covariates used in the minimisation algorithm (qualifying ICH location, time since ICH symptom onset, antiplatelet drug(s) that patient’s physician would start if allocated, participant age at randomisation, predicted 6-month outcome)
- Sex
- Ethnicity
- Functional status
- Modified Rankin scale (mRS) score
- Co-morbidities
- Antithrombotic drugs taken before ICH
- Timing of key events (symptom onset to randomisation, symptom onset to earliest imaging study, earliest imaging study to randomisation, symptom onset to sub-study MRI (if applicable), and sub-study MRI to randomisation (if applicable))
- Characteristics of the ICH and brain on diagnostic imaging and sub-study MRI (if applicable)
  - The RESTART imaging panel’s report of CT diagnostic imaging will describe the baseline characteristics of the trial population’s brains (e.g. old vascular lesions, periventricular lucencies and atrophy) and ICH (e.g. location, intraventricular extension, subarachnoid extension, ICH volume and finger-like projections [24])
  - The RESTART imaging panel’s report of MRI sub-study protocol-compliant imaging will describe the baseline characteristics of the participants’ brains (e.g. brain microbleed presence/location and burden, old infarcts or haemorrhages, superficial siderosis, white matter hyperintensities, and atrophy) and ICH (e.g. location, intraventricular extension, subarachnoid extension, and ICH volume). In the MRI sub-study, the focus of our hypothesis will be on testing whether the presence, number or location of brain microbleeds modifies the effect of antiplatelet drugs on the primary outcome, adjusted for the same covariates used in the primary analysis of the entire RCT. For dichotomous analysis, the number of brain microbleeds will be split as 0 or 1 versus 2 or more and for categorical analysis the split will be 0 or 1 versus 2 to 4 versus 5 or more. Brain microbleed location will be grouped as strictly lobar versus other

### Outcomes

We collected symptomatic outcomes during follow-up without explicit definitions in order to minimise the complexity of the reporting process for participants, their general practitioners, carers and investigators. During the outcome event adjudication process, we were guided by the principles and definitions described by the Standardised Data Collection for Cardiovascular Trials Initiative [25]. We classify events as fatal (if they were followed by death within 30 days) or non-fatal (if they were not followed by death within 30 days).

- Fatal or non-fatal radiographically or pathologically proven recurrent symptomatic ICH. We define this as the abrupt onset of headache, altered level of consciousness, or focal neurological deficit, anatomically referable to a focal collection of blood predominantly located within the brain parenchyma (diagnosed on brain imaging or at autopsy), which was not attributable to prior trauma or haemorrhagic transformation of an ischaemic stroke. This also applies when neurological deterioration occurs with radiographic or pathological evidence of ICH volume growth early after the qualifying ICH (due to either haematoma expansion or re-bleeding)
- Symptomatic spontaneous or traumatic extradural haemorrhage, subdural haemorrhage, subarachnoid haemorrhage, or intraventricular haemorrhage (not accompanying spontaneous ICH)
- Symptomatic major extracranial haemorrhage (requiring transfusion or endoscopic treatment or surgery, or resulting in death within 30 days, i.e. Bleeding Academic Research Consortium definition for bleeding types 3–5 [26])
- Transient ischaemic attack
- Ischaemic stroke
- Acute coronary syndrome (restricted to STEMI and non-STEMI myocardial infarction, but not hospitalisation for unstable angina)
- Peripheral arterial occlusion
- Mesenteric ischaemia
- Retinal arterial occlusion
- Deep vein thrombosis
- Pulmonary embolism
- Revascularisation procedures (carotid, percutaneous coronary intervention or peripheral arterial intervention)
- Sudden cardiac death with symptoms suggestive of myocardial ischaemia (type 3) or evidence of arrhythmia (i.e. sudden cardiac death [25])
- Symptomatic stroke of uncertain sub-type
  - Non-fatal stroke, with brain imaging performed too late to distinguish ICH from cerebral infarction
  - Rapidly fatal stroke, but without radiographic or pathological confirmation
- Deaths not due to fatal major vascular events above
  - Other cardiovascular deaths (e.g. due to heart failure, cardiovascular procedures, cardiovascular haemorrhage)
  - Non-cardiovascular deaths
  - Deaths of undetermined cause
- Annual ratings of participant function completed by participants or their carer using the simplified mRS postal questionnaire [27, 28], or structured telephone interview with non-responders to the postal questionnaire [29]

The primary outcome is fatal or non-fatal radiographically or pathologically proven recurrent symptomatic ICH. The two key secondary outcomes are a composite of all major haemorrhagic events and a composite of all major vaso-occlusive events (including revascularisation procedures):

- The composite secondary outcome of ‘all major haemorrhagic events’ includes the following fatal or non-fatal symptomatic events (that are ‘major’ because of their usual need for hospitalisation and influence on outcome and antithrombotic treatment):
  - Radiographically or pathologically proven recurrent symptomatic ICH (the primary outcome)
  - Other forms of symptomatic spontaneous or traumatic intracranial haemorrhage, including radiographically or pathologically proven spontaneous or traumatic extradural haemorrhage, subdural haemorrhage, subarachnoid haemorrhage or intraventricular haemorrhage (not accompanying spontaneous ICH)
  - Extracranial haemorrhage at any site requiring transfusion/endoscopic treatment/surgery, or resulting in death
- The composite secondary outcome of ‘all major vaso-occlusive events (including revascularisation procedures)’ includes the following fatal or non-fatal symptomatic events (that are ‘major’ because of their usual need for hospitalisation and influence on outcome and antithrombotic treatment; therefore, we will not include transient ischaemic attack or retinal artery occlusion) or revascularisation procedures:
  - Fatal or non-fatal vaso-occlusive events, including ischaemic stroke, acute coronary syndrome, mesenteric ischaemia or peripheral arterial occlusion, deep vein thrombosis, or pulmonary embolism
  - Carotid, coronary or peripheral arterial revascularisation procedures

The composite secondary outcome of ‘all major haemorrhagic or vaso-occlusive events’ combines the two composites defined above. For completeness, the corresponding analysis will also be performed for each composite outcome event proposed in the trial protocol. The interpretation of the trial findings will respect this pre-specified hierarchy of primary outcome, key secondary outcomes and other secondary outcomes, and no formal adjustment will be made to significance levels to allow for multiplicity.

We will summarise the total numbers of the primary and secondary outcomes during follow-up, as well as the number of first events of each type during follow-up, overall and by treatment group.

Serious adverse events are reported in RESTART if they are neither an outcome event nor an expected complication of stroke. Serious adverse events will be grouped by body system and for each event and each grouped set of events, we will quantify the number of events and the number of individuals experiencing at least one such event per allocated treatment group.

We will report adherence (at last follow-up before the occurrence of the first outcome event) to the allocated antiplatelet treatment strategy (start vs. avoid antiplatelet drugs) and use of anticoagulant drugs descriptively per treatment group at discharge and at each annual follow-up. Because blood pressure (BP) control is a potential confounder of the frequency of the primary outcome, the use of BP-lowering drugs at discharge after randomisation and at each annual follow-up will be reported (along with a summary of the available BPs of participants by treatment group).

Any change, divergence or departure from the trial design or procedures defined in the protocol or Good Clinical Practice are identified and recorded as a deviation (if it does not significantly affect a participant’s rights, safety or well-being, or trial outcomes), or a violation (if the deviation may potentially significantly impact the completeness, accuracy and/or reliability of the trial data or that may significantly affect a patient’s rights, safety or well-being). We will list all protocol deviations and violations, but no formal statistical testing will be performed.

### Methods of analysis

In general terms, we will present categorical data using counts and percentages, continuous variables using the mean, median, standard deviation (SD), minimum, maximum, inter quartile points at 25% and 75% (Q1 and Q3), and number of patients with an observation (n). We will estimate the survival function per treatment group using a Kaplan–Meier survival analysis of time to first outcome event during all available follow-up after randomisation. We will censor follow-up at death (unrelated to an outcome event), last available follow-up or voluntary withdrawal from the trial. We will compare the survival function in the two trial arms using a Cox proportional hazards regression model, adjusting for all the covariates included in the minimisation algorithm (qualifying ICH location of lobar versus non-lobar; time since ICH symptom onset of 1–6 days versus 7–30 days versus over 30 days; antiplatelet drug(s) that the patient’s clinician would start if allocated (aspirin alone vs. any other regimen); participant’s age at randomisation (< 70 years vs. ≥ 70 years); predicted probability of a good 6-month outcome (< 0.15 vs. ≥ 0.15)), and presenting the result as an estimated adjusted hazard ratio with its corresponding 95% CI. We will also report the unadjusted estimate of the hazard ratio and its corresponding 95% CI, together with the result of the logrank test. We will estimate the absolute difference in event rates at 1, 2, 3 and 4 years from the Kaplan–Meier analysis.

We will conduct two exploratory sensitivity analyses of our primary analysis, by adding the following secondary outcomes to the primary outcome in the following order, to account for the possibility that some fatalities and non-fatal neurological events without adequate investigation may be recurrent ICH:

- Symptomatic stroke of uncertain sub-type
- Deaths of undetermined cause

We will conduct a sensitivity analysis of the secondary outcomes to reflect the cumulative incidence of major haemorrhagic or vaso-occlusive events, using re-randomisation tests [30].

We will perform sub-group analyses for the primary outcome and the key secondary outcomes including (1) all major haemorrhagic events, (2) all major vaso-occlusive events (including revascularisation procedures), and (3) the composite of all major haemorrhagic or vaso-occlusive events. We will analyse heterogeneity of treatment effect between sub-groups using statistical tests of interaction. These analyses will be performed by including an interaction term between treatment group and the relevant covariate in the Cox proportional hazards regression model. Time since ICH will be dichotomised in view of the distribution of outcomes in the whole trial dataset. Should any of these adjusted regression models fail to converge, then the corresponding unadjusted analysis will be reported instead. We have specified a priori the following dichotomous sub-groups for the primary analysis:

- The five covariates used in the minimisation algorithm
  - Qualifying ICH location (lobar vs. non-lobar)
  - Time since ICH symptom onset (two subgroups will be defined based on the time from onset being above or below the median time observed in the trial)
  - Antiplatelet drug(s) that the patient’s clinician would start if allocated (aspirin alone vs. any other regimen)
  - Participant’s age at randomisation (< 70 years vs. ≥ 70 years)
  - Predicted probability of a good 6-month outcome (< 0.15 vs. ≥ 0.15)
- Pre-ICH antithrombotic drug regimen (antiplatelet vs. anticoagulant)
- History of atrial fibrillation documented as a co-morbidity at randomisation (yes vs. no)

We will perform a separate analysis for each annual assessment of the mRS score. The analysis at year ‘x’ will be restricted to participants who were randomised at least ‘x’ years prior to study close, to avoid including early deaths in the relevant follow-up year when the corresponding surviving recruits would not have had the potential to be assessed. The analysis will comprise a tabulation of mRS by randomised group, with the formal analysis being based on a Mann–Whitney test. Participants’ type of domicile will be described as categorised on the discharge form and each annual participant questionnaire.

### Statistical principles

We will use available data and not impute data with regard to missing values or withdrawals. We will not perform formal statistical tests of baseline characteristics or adherence by treatment group. All applicable statistical tests will be two-sided and will be performed using a 5% significance level, leading to 95% (two-sided) confidence intervals, unless otherwise specified. We will assess the proportional hazards assumption graphically; if there is strong evidence of violation of the assumption, the impact on the analysis will be assessed by comparing the results of the pre-specified analysis with the results obtained using the restricted survival time approach [31].

### Statistical software

The un-blinded trial statistician at the Edinburgh Clinical Trials Unit (ECTU) will perform the statistical programming and analysis to produce all summary tables and figures using the statistical package SAS (v9.3 or a more recent version).

### Quality control

Before database lock and un-blinding, a full statistical report will be produced based on dummy randomisation codes to allow for checking of the data and the proposed summaries/analyses. Isolated data errors detected in the database as a result of quality control checks that are deemed significant will be submitted for enquiry to the trial manager or designee. Systematic data errors in the data reporting will be investigated further; the data will be corrected if necessary, and the appropriate table then re-checked. A random selection of unique analysis and summary tables will be checked using manual methods (i.e. comparison of results in the table to results calculated by a calculator, spreadsheet, database output or any alternative summarisation tool). Checks of statistical analyses will be performed by peer review of program code, log and output. Additionally, the primary outcomes analysis will be replicated independently by a second statistician.

### Administrative information

The published RESTART protocol reflected version 7.0, created on 23 December 2015 [17]. The final amendment that was made to the protocol (version 8.0, created on 19 September 2017) was an update to the reference safety information used for clopidogrel. Standard operating procedures produced by the sponsor (http://www.accord.scot/research-access/resources-researchers/sop) guided trial conduct. ECTU standard operating procedures guided the content and format of the final version of the statistical analysis plan (https://www.ed.ac.uk/usher/edinburgh-clinical-trials/supporting-trials/governance/standard-operating-procedures). This published statistical analysis plan complies with relevant reporting standards and reflects version 1.6 of the statistical analysis plan (see Additional files 1 and 2), which was written and signed by the blinded trial statistician (GDM), was counter-signed by the chief investigator (RA-SS), and included a history of revisions and their timings [32]. The definitions of outcome events have been expanded upon in this published version of the statistical analysis plan. The funder and sponsor did not require a data management plan, but plans for data sharing were described in the published protocol [17]. The sponsor delegated responsibility for maintaining the trial master file to the trial management group (2013/W/NEU/04/TMF), who keep it in DataStore, which is the University of Edinburgh’s file store for active research data (https://www.ed.ac.uk/information-services/research-support/research-data-service/working-with-data/data-storage). The un-blinded trial statistician maintains a paper copy of the statistical master file, which is held securely within ECTU.

## Discussion

This published version of the final RESTART statistical analysis plan, which we have reported according to recent guidelines [32], describes details of our approach to describing the trial population, the statistical principles we will apply to the analysis population, and the analyses we will undertake. We submitted this statistical analysis plan before the end of follow-up and data lock, with one minor modification after the end of follow-up but before database lock and un-blinding, in view of the distribution of outcomes in the entire analysis population. We standardised outcome event definitions before un-masking the trial steering committee to the results [25, 26]. This report should provide reassurance about protection of the trial from selective outcome reporting and data-driven analyses.

The results of this trial will help to estimate, for the first time, the effects of antiplatelet drugs on recurrent ICH. This will provide information about the likely net effects of antiplatelet drugs after ICH, and whether a larger RCT will be required to estimate effects on all major vascular events or functional outcome.

## Trial status

Recruitment ended on 31 May 2018. Follow-up ended on 30 November 2018. Data lock occurred on 29 January 2019, after which the un-blinded trial statistician conducted the final analyses according to this statistical analysis plan. The current protocol is version 8.0, created 19 September 2017 (all protocol updates since version 3.0, created 1 February 2013, which was implemented before randomisation began, have been approved by the sponsor and research ethics committee and also communicated to investigators and trial registries). This manuscript is based on version 1.7 of the RESTART statistical analysis plan, finalised on 25 January 2019.

## Additional files

Additional file 1: SAP checklist. Reporting checklist: recommended items to address in a clinical trial Statistical Analysis Plan [32]. (DOCX 38 kb)

Additional file 2: 2019_01_25_RESTART SAP_v1.7_signed. Final version of the RESTART statistical analysis plan. (PDF 609 kb)

## Abbreviations

BP: blood pressure
CT: computed tomography
ECTU: Edinburgh Clinical Trials Unit
ICH: intracerebral haemorrhage
MRI: magnetic resonance imaging
mRS: modified Rankin scale
RCT: randomised controlled trial
RESTART: REstart or STop antithrombotics randomised trial

## References

[1] Poon MT, Fonville AF, Al-Shahi Salman R (2014). Long-term prognosis after intracerebral haemorrhage: systematic review and meta-analysis. J Neurol Neurosurg Psychiatry.

[2] Al-Shahi Salman R, Frantzias J, Lee RJ, Lyden PD, Battey TWK, Ayres AM, Goldstein JN, Mayer SA, Steiner T, Wang X (2018). Absolute risk and predictors of the growth of acute spontaneous intracerebral haemorrhage: a systematic review and meta-analysis of individual patient data. Lancet Neurol.

[3] Steiner T, Al-Shahi Salman R, Beer R, Christensen H, Cordonnier C, Csiba L, Forsting M, Harnof S, Klijn CJ, Krieger D (2014). European Stroke Organisation (ESO) guidelines for the management of spontaneous intracerebral hemorrhage. Int J Stroke.

[4] Hemphill JC, Greenberg SM, Anderson CS, Becker K, Bendok BR, Cushman M, Fung GL, Goldstein JN, Macdonald RL, Mitchell PH (2015). Guidelines for the management of spontaneous intracerebral hemorrhage: a guideline for healthcare professionals from the American Heart Association/American Stroke Association. Stroke.

[5] van Asch CJ, Luitse MJ, Rinkel GJ, van der Tweel I, Algra A, Klijn CJ (2010). Incidence, case fatality, and functional outcome of intracerebral haemorrhage over time, according to age, sex, and ethnic origin: a systematic review and meta-analysis. Lancet Neurol.

[6] Balami JS, Buchan AM (2012). Complications of intracerebral haemorrhage. Lancet Neurol.

[7] Chapman N, Huxley R, Anderson C, Bousser MG, Chalmers J, Colman S, Davis S, Donnan G, MacMahon S, Neal B (2004). Effects of a perindopril-based blood pressure-lowering regimen on the risk of recurrent stroke according to stroke subtype and medical history: the PROGRESS Trial. Stroke.

[8] PROGRESS Collaborative Group (2001). Randomised trial of a perindopril-based blood-pressure-lowering regimen among 6,105 individuals with previous stroke or transient ischaemic attack. Lancet.

[9] Ding X, Liu X, Tan C, Yin M, Wang T, Liu Y, Mo L, Wei X, Tan X, Deng F (2018). Resumption of antiplatelet therapy in patients with primary intracranial hemorrhage-benefits and risks: a meta-analysis of cohort studies. J Neurol Sci.

[10] Flynn RW, MacDonald TM, Murray GD, MacWalter RS, Doney AS (2010). Prescribing antiplatelet medicine and subsequent events after intracerebral hemorrhage. Stroke.

[11] Biffi A, Halpin A, Towfighi A, Gilson A, Busl K, Rost N, Smith EE, Greenberg MS, Rosand J, Viswanathan A (2010). Aspirin and recurrent intracerebral hemorrhage in cerebral amyloid angiopathy. Neurology.

[12] Chong BH, Chan KH, Pong V, Lau KK, Chan YH, Zuo ML, Lui WM, Leung GK, Lau CP, Tse HF (2012). Use of aspirin in Chinese after recovery from primary intracranial haemorrhage. Thromb Haemost.

[13] Teo KC, Lau GK, Mak RH, Leung HY, Chang RS, Tse MY, Lee R, Leung GK, Siu DC, Cheung RT (2017). Antiplatelet resumption after antiplatelet-related intracerebral hemorrhage: a retrospective hospital-based study. World Neurosurg.

[14] Gonzalez-Perez A, Gaist D, de Abajo FJ, Saez ME, Garcia Rodriguez LA (2017). Low-dose aspirin after an episode of haemorrhagic stroke is associated with improved survival. Thromb Haemost.

[15] Glasziou P, Chalmers I, Rawlins M, McCulloch P (2007). When are randomised trials unnecessary? Picking signal from noise. BMJ.

[16] Perry LA, Berge E, Bowditch J, Forfang E, Ronning OM, Hankey GJ, Villanueva E, Al-Shahi Salman R (2017). Antithrombotic treatment after stroke due to intracerebral haemorrhage. Cochrane Database Syst Rev.

[17] Al-Shahi Salman R, Dennis MS, Murray GD, Innes K, Drever J, Dinsmore L, Williams C, White PM, Whiteley WN, Sandercock PAG (2018). The REstart or STop Antithrombotics Randomised Trial (RESTART) after stroke due to intracerebral haemorrhage: study protocol for a randomised controlled trial. Trials.

[18] Counsell C, Dennis M, McDowall M, Warlow C (2002). Predicting outcome after acute and subacute stroke: development and validation of new prognostic models. Stroke.

[19] Peto R, Pike MC, Armitage P, Breslow NE, Cox DR, Howard SV, Mantel N, McPherson K, Peto J, Smith PG (1976). Design and analysis of randomized clinical trials requiring prolonged observation of each patient. I. Introduction and design. Br J Cancer.

[20] Haybittle JL (1971). Repeated assessment of results in clinical trials of cancer treatment. Br J Radiol.

[21] Peto R, Pike MC, Armitage P, Breslow NE, Cox DR, Howard SV, Mantel N, McPherson K, Peto J, Smith PG (1977). Design and analysis of randomized clinical trials requiring prolonged observation of each patient. II. analysis and examples. Br J Cancer.

[22] Maxwell AE, MacLeod MJ, Joyson A, Johnson S, Ramadan H, Bellfield R, Byrne A, McGhee C, Rudd A, Price F (2017). Reasons for non-recruitment of eligible patients to a randomised controlled trial of secondary prevention after intracerebral haemorrhage: observational study. Trials.

[23] Clark TG, Altman DG, De Stavola BL (2002). Quantification of the completeness of follow-up. Lancet.

[24] Rodrigues MA, Samarasekera N, Lerpiniere C, Humphreys C, McCarron MO, White PM, Nicoll JAR, Sudlow CLM, Cordonnier C, Wardlaw JM (2018). The Edinburgh CT and genetic diagnostic criteria for lobar intracerebral haemorrhage associated with cerebral amyloid angiopathy: model development and diagnostic test accuracy study. Lancet Neurol.

[25] Hicks KA, Mahaffey KW, Mehran R, Nissen SE, Wiviott SD, Dunn B, Solomon SD, Marler JR, Teerlink JR, Farb A (2018). 2017 Cardiovascular and stroke endpoint definitions for clinical trials. Circulation.

[26] Mehran R, Rao SV, Bhatt DL, Gibson CM, Caixeta A, Eikelboom J, Kaul S, Wiviott SD, Menon V, Nikolsky E (2011). Standardized bleeding definitions for cardiovascular clinical trials: a consensus report from the Bleeding Academic Research Consortium. Circulation.

[27] Bruno A, Shah N, Lin C, Close B, Hess DC, Davis K, Baute V, Switzer JA, Waller JL, Nichols FT (2010). Improving modified Rankin Scale assessment with a simplified questionnaire. Stroke.

[28] Dennis M, Mead G, Doubal F, Graham C (2012). Determining the modified Rankin score after stroke by postal and telephone questionnaires. Stroke.

[29] Bruno A, Akinwuntan AE, Lin C, Close B, Davis K, Baute V, Aryal T, Brooks D, Hess DC, Switzer JA (2011). Simplified modified rankin scale questionnaire: reproducibility over the telephone and validation with quality of life. Stroke.

[30] Ford I, Murray H, McCowan C, Packard CJ (2016). Long-term safety and efficacy of lowering low-density lipoprotein cholesterol with statin therapy: 20-year follow-up of West of Scotland Coronary Prevention Study. Circulation.

[31] Royston P, Parmar MK (2011). The use of restricted mean survival time to estimate the treatment effect in randomized clinical trials when the proportional hazards assumption is in doubt. Stat Med.

[32] Gamble C, Krishan A, Stocken D, Lewis S, Juszczak E, Dore C, Williamson PR, Altman DG, Montgomery A, Lim P (2017). Guidelines for the content of statistical analysis plans in clinical trials. JAMA.

