# Supplementary material for: The REstart or STop Antithrombotics Randomised Trial (RESTART) after stroke due to intracerebral haemorrhage: statistical analysis plan for a randomised controlled trial
Source: Trials. 2019 Mar 25;20:183. doi: 10.1186/s13063-019-3270-2 (PMC6434884; doi:10.1186/s13063-019-3270-2)
Supplement: Supplementary file 1 — SAP checklist. Reporting checklist: recommended items to address in a clinical trial Statistical Analysis Plan [32]. (DOCX 38 kb) [file 13063_2019_3270_MOESM1_ESM.docx]

| **Section/Item** | **Index** | **Description** | **Page** |
| --- | --- | --- | --- |
| **Section 1: Administrative Information** | |  |  |
| Title and Trial registration | 1a | Descriptive title that matches the protocol, with ‘Statistical analysis plan’ either as a fore runner or sub title, and trial acronym ( if applicable) | 1 |
|  | 1b | Trial registration number | 3 |
| SAP Version | 2 | SAP version number with dates | 21 |
| Protocol Version | 3 | Reference to version of Protocol being used | 21 |
| SAP Revisions | 4a | SAP revision history | See attached file SAP V1.6_2018-09-18_signed.pdf |
|  | 4b | Justification for each SAP revision | See attached file SAP V1.6_2018-09-18_signed.pdf |
|  | 4c | Timing of SAP revisions in relation to interim analyses etc. | See attached file SAP V1.6_2018-09-18_signed.pdf |
| Roles and Responsibility | 5 | Names, affiliations, and roles of SAP contributors | 1, 22-23 |
| Signatures of: | 6a | - Person writing the SAP | See attached file SAP V1.6_2018-09-18_signed.pdf |
|  | 6b | - Senior statistician responsible |  |
|  | 6c | - Chief investigator/clinical lead |  |
| **Section 2: Introduction** |  |  |  |
| Background and rationale | 7 | Synopsis of trial background and rationale including a brief description of research question and brief justification for undertaking the trial | 5-6 |
| Objectives | 8 | Description of specific objectives or hypotheses | 6-7 |
| **Section 3: Study Methods** |  |  |  |
| Trial design | 9 | Brief description of trial design including type of trial (e.g. parallel group, multiarm, crossover, factorial) and allocation ratio and may include brief description of interventions | 6-7 |
| Randomization | 10 | Randomization details e.g. whether any minimization or stratification occurred (including stratifying factors used or the location of that information if it is not held within the SAP) | 6 |
| Sample size | 11 | Full sample size calculation or reference to sample size calculation in protocol (instead of replication in SAP) | 6 |
| Framework | 12 | Superiority, equivalence or non-inferiority hypothesis testing framework, including which comparisons will be presented on this basis | 6 |
| Statistical Interim analyses and stopping guidance | 13a | Information on interim analyses specifying what interim analyses will be carried out and listing of time points | 7 |
|  | 13b | Any planned adjustment of the significance level due to interim analysis | 7 |
|  | 13c | Details of guidelines for stopping the trial early | 7 |
| Timing of final analysis | 14 | Timing of final analysis e.g. all outcomes analyzed collectively or timing stratified by planned length of follow-up | 16 |
| Timing of outcome assessments | 15 | Time points at which the outcomes are measured including visit ‘windows’ | 8 |
| **Section 4: Statistical Principles** | | |  |
| Confidence intervals and p-values | 16 | Level of statistical significance | 18 |
|  | 17 | Description and rationale for any adjustment for multiplicity, and if so, detailing how the type 1 error is to be controlled | N/A |
|  | 18 | Confidence intervals (CI) to be reported | 18 |
| Adherence and Protocol Deviations | 19a | Definition of adherence to the intervention and how this is assessed including extent of exposure | 15 |
|  | 19b | Description of how adherence to the intervention will be presented | 15 |
|  | 19c | Definition of protocol deviations for the trial | 15 |
|  | 19d | Description of which protocol deviations will be summarized | 15 |
| Analysis populations | 20 | Definition of analysis populations e.g. Intention to treat, Per protocol, complete case, safety | 8 |
| **Section 5:Trial Population** |  |  |  |
| Screening data | 21 | Reporting of screening data (if collected) to describe representativeness of trial sample | 7 |
| Eligibility | 22 | Summary of eligibility criteria | 8 |
| Recruitment | 23 | Information to be included in the CONSORT flow diagram | 7-8 |
| Withdrawal/Follow up | 24a | Level of withdrawal e.g. from intervention and/or from follow-up | 8 |
|  | 24b | Timing of withdrawal/lost to follow up data | 16 |
|  | 24c | Reasons and details of how withdrawal/lost to follow up data will be presented | 8 |
| Baseline patient characteristics | 25a | List of baseline characteristics to be summarized | 9-11 |
|  | 25b | Details of how baseline characteristics will be descriptively summarized | 16 |
| **Section 6: Analysis** |  |  |  |
| Outcome definitions |  | List and describe each primary and secondary outcome including details of: | 11-14 |
|  | 26a | - specification of outcomes and timings. If applicable include the order of importance of primary or key secondary endpoints (e.g. order in which they will be tested) | 14 |
|  | 26b | - specific measurement and units (e.g. glucose control hbA1c (mmol/mol or %)) | N/A |
|  | 26c | - any calculation or transformation used to derive the outcome (e.g. change from baseline, QoL score, time to event, logarithm etc) | N/A |
| Analysis methods | 27a | - what analysis method will be used, and how the treatment effects will be presented | 16 |
|  | 27b | - any adjustment for covariates | 16 |
|  | 27c | - methods used for assumptions to be checked for statistical methods | 18-19 |
|  | 27d | -   details of alternative methods to be used if distributional assumptions do not hold e.g. normality, proportional hazards etc | 18-19 |
|  | 27e | - any planned sensitivity analyses for each outcome where applicable | 16-17 |
|  | 27f | - any planned subgroup analyses for each outcome including how subgroups are defined | 17-18 |
| Missing data | 28 | Reporting and assumptions/statistical methods to handle missing data (e.g. multiple imputation) | 18 |
| Additional analyses | 29 | Details of any additional statistical analyses required e.g. Complier-average causal effect (CACE^23^) analysis | N/A |
| Harms | 30 | Sufficient detail on summarizing safety data e.g. information on severity, expectedness and causality; details of how adverse events (AE's) are coded or categorized; how AE data will be analyzed, i.e. grade 3/4 only, incidence case analysis, intervention emergent analysis | 15 |
| Statistical Software | 31 | Details of statistical packages to be used to carry out analyses | 19 |
| References | 32a | References to be provided for non-standard statistical methods | N/A |
|  | 32b | Reference to Data Management Plan | 20 |
|  | 32c | Reference to the Trial Master File and Statistical Master File | 20 |
|  | 32d | Reference to other standard operating procedures ( SOPs) or documents to be adhered to. | 20 |
