# Supplementary material for: The REstart or STop Antithrombotics Randomised Trial (RESTART) after stroke due to intracerebral haemorrhage: statistical analysis plan for a randomised controlled trial
Source: Trials. 2019 Mar 25;20:183. doi: 10.1186/s13063-019-3270-2 (PMC6434884; doi:10.1186/s13063-019-3270-2)
Supplement: Supplementary file 2 — 2019_01_25_RESTART SAP_v1.7_signed. Final version of the RESTART statistical analysis plan. (PDF 609 kb) [file 13063_2019_3270_MOESM2_ESM.pdf]

REstart or STop Antithrombotics Randomised Trial

STATISTICAL ANALYSIS PLAN

**ISRCTN Number**	ISRCTN71907627
**EudraCT Number**	2012-003190-26
**Funder:**	British Heart Foundation
**Sponsors**	The University of Edinburgh and NHS Lothian
**Version:**	1.7
**Date:**	25th January 2019
**Chief Investigator:**	Professor Rustam Al-Shahi Salman<br>rustam.al-shahi@ed.ac.uk
**Author:**	Professor Gordon Murray<br>Trial Statistician<br>gordon.murray@ed.ac.uk

Professor Gordon Murray<br>(University of Edinburgh)	25/01/2019<br>Date
Professor Rustam Al-Shahi Salman<br>(University of Edinburgh)	25/01/2019<br>Date

Document Version History

Version Number	Reason for Update	Updated By:	Date
1.00	Creation of new statistical analysis plan	Gordon Murray	8th October 2015
1.01	Revised ahead of circulation to TSC for discussion, with initial input from CI	Gordon Murray	14th October 2015
1.1	Revised following discussion with TSC	Gordon Murray	10th December 2015
1.2	Revised to reflect Version 7.0 of the protocol and to take account of the funding extension which allows the trial to run until 28th February 2019	Gordon Murray	19th April 2016
1.3	Revised following discussions with the TSC Chair	Gordon Murray/ Rustam Al-Shahi Salman	26th September 2017
1.4	Revised following discussions with the TSC	Gordon Murray	16th October 2017
1.5	Revised to reflect Version 8.0 of the protocol, and to be compliant with published guidelines on the content of Statistical Analysis Plans (*JAMA* 2017;318(23):2337-2343)	Gordon Murray	29th March 2018
1.6	Revised to reflect the primary sources of outcome ascertainment that will be used by the trial, analyses that will be included in the primary report of the main trial, and the imaging analyses that will be reported in a separate publication.	Rustam Al-Shahi Salman	18th September 2018
1.7	Revised the categorisation of time since ICH for analysis of the MRI sub-study in view of the distribution of outcomes in the whole trial dataset and specified the approach to be followed should any of the multivariate analysis models used with the MRI sub-study data fail to converge (before database lock and un-blinding to treatment allocation)	Rustam Al-Shahi Salman / Gordon Murray	25th January 2019

1 Table of contents

1 Table of contents 3

2 List of abbreviations 5

3 Introduction 6

3.1 Responsibilities 6
3.2 Definitions 6

4 Study design 6

4.1 Brief Description 6

5 Overall statistical principles 7

5.1 Handling of missing data 7
5.2 Quality control (QC) of summary tables and statistical analysis 7
5.2.1 QC/Validation - Summary Tables 7
5.2.2 QC/Validation - Statistical Analysis 8
5.3 Un-blinding procedures 8

6 Population for analysis 8

6.1 Intention-to-treat population 8
6.2 Datasets for analysing the main study and the imaging sub-studies 8

7 List of analyses to be performed 8

7.1 Recruitment and retention 8
7.2 Baseline data: demographics, baseline/clinical characteristics 8
7.3 Protocol deviations/violations 9
7.4 Adherence to allocated treatment 9
7.5 Co-interventions 9
7.6 Analysis of outcomes 9
7.7 Primary outcome 9
7.7.1 Sensitivity analyses 10
7.8 Secondary outcomes 10
7.8.1 All serious haemorrhagic events 10
7.8.2 All serious vaso-occlusive events (including revascularisation procedures) 10
7.8.3 Composite of all serious haemorrhagic or vaso-occlusive events 10
7.8.4 Sensitivity analysis 11
7.8.5 Annual assessment of modified Rankin Scale (mRS) 11
7.9 Sub-group analyses 11
7.10 Serious adverse events 11
7.11 Imaging sub-studies 11

8 Data Sharing 12

Appendix 1: Outcome measures specified in the protocol 13

Primary 13
Secondary 13
Annual ratings of participant function completed by participant or their carer 13

Appendix 2: Statistical extracts from the protocol 14

Sample size calculation 14
Proposed analyses 14
Primary analysis 15
Secondary analyses 15
Sub-group analyses 15
Brain MRI sub-study 16

2 List of abbreviations

BP	Blood pressure
CI	Confidence interval
CONSORT	CONsolidated Standards of Reporting Trials
CT	Computed tomography
ECTU	Edinburgh Clinical Trials Unit
GP	General practitioner
ICH	Intracerebral haemorrhage
ITT	Intention-to-treat
MRI	Magnetic resonance imaging
mRS	Modified Rankin Scale
n	Number of patients with an observation
Q1	First (lower) quartile
Q3	Third (upper) quartile
QC	Quality control
RCT	Randomised controlled trial
RESTART	REstart or STop Antithrombotics Randomised Trial
RR	Relative risk
SAE	Serious adverse event
SAP	Statistical Analysis Plan
SAS	Statistical Analysis System
SD	Standard deviation
SOP	Standard Operating Procedure
TSC	Trial Steering Committee

3 Introduction

This document details the criteria to be used for the definition of the analysis populations and the statistical methodology for analysis of the Restart or Stop Antithrombotics Randomised Trial (RESTART). This document has been compiled according to Edinburgh Clinical Trials Unit (ECTU) standard operating procedure (SOP) “Statistical and Analysis Plans”. This document has been written based on information contained in the final study protocol dated 19th September 2017, Version 8.0.

For ease of reference, two appendices contain extracts from the study protocol. Appendix 1 lists the study outcomes as set out in the protocol and Appendix 2 sets out the statistical aspects, comprising the sample size justification and an outline of the proposed statistical analyses.

3.1 Responsibilities

The statistician at ECTU will be responsible for the production of the following items using ECTU SOPs: summary tables, formal statistical analysis and the statistical report.

3.2 Definitions

Throughout the reporting of the study, the studied interventions will be reported as ‘start antiplatelet drugs’ versus ‘avoid antiplatelet drugs’, and will be collectively referred as the treatment groups.

4 Study design

4.1 Brief Description

RESTART is a randomised controlled trial (RCT) involving two parallel groups, designed to help assess whether patients who survive a stroke due to spontaneous intracerebral haemorrhage (ICH) which developed while the patient was taking antithrombotic drugs for the prevention of vaso-occlusive disease should start antiplatelet drugs for continued secondary prevention of vaso-occlusive disease.

Full inclusion/exclusion criteria are set out in the protocol, but in summary patients are: aged 18 years or older; have suffered a spontaneous ICH not attributable to preceding head injury; have taken an antithrombotic drug for the prevention of vaso-occlusive disease before the onset of the qualifying ICH; and the patient and their physician are both uncertain about whether to start or avoid antiplatelet drugs.

The primary objective of the RCT is to estimate the relative and absolute effects of antiplatelet drugs on the risk of recurrent symptomatic ICH associated with a policy of starting antiplatelet drugs after the acute phase of spontaneous ICH. The aim is to recruit 720 patients (360 per treatment group). Since the primary objective of the trial is to estimate a treatment effect, the trial does not fit into a conventional superiority, equivalence or noninferiority hypothesis testing framework.

RESTART collects all participants’ diagnostic brain imaging (usually computed tomography [CT], but sometimes magnetic resonance imaging [MRI] alone) that diagnosed ICH before randomisation. An imaging sub-study was also conducted involving brain MRI to test for an interaction between the presence of brain microbleeds (which are one of many imaging biomarkers of cerebral small vessel disease) and the effects of antiplatelet drugs. The aim was to recruit at least 550 of the 720 RESTART participants into the MRI sub-study.

RESTART follows participants via: clinic/hospital discharge forms; annual postal/telephone questionnaires to participants and their general practitioners (GPs); and *ad hoc* reports from GPs, participants, carers, or research staff sites. RESTART will request, obtain and examine linked data on participants held by administrative datasets (death certificates and hospital admissions), but because all of these sources of data are not available in all of the UK countries participating in RESTART, administrative data will not be a primary source of ascertainment of information about outcome events in RESTART.

There are no formal planned interim analyses. Accumulating data from the trial are being monitored by an independent Data Monitoring Committee. The stopping guideline is based on the 'overwhelming evidence' principle, and hence there is no need to adjust the final significance level of this RCT.

5 Overall statistical principles

The statistician at ECTU will perform the statistical programming and analysis to produce all summary tables and figures using the statistical package SAS (v9.3 or a more recent version).

In general terms, categorical data will be presented using counts and percentages, whilst continuous variables will be presented using the mean, median, standard deviation (SD), minimum, maximum, inter quartile points at 25% and 75% (Q1 and Q3) and number of patients with an observation (n).

All applicable statistical tests will be 2-sided and will be performed using a 5% significance level, leading to 95% (2-sided) confidence intervals (CIs), unless otherwise specified.

All analyses will follow the "intention-to-treat" principle.

5.1 Handling of missing data

There will be no imputation for the data with regard to missing values or withdrawals for the statistical summaries and statistical analysis unless otherwise specified.

Some of the covariates specified for the statistical analyses are required to inform the minimisation algorithm, and so there should be no missing data for these baseline covariates. Strenuous efforts are being made to obtain virtually complete outcome data.

Any required/applicable imputation of data will be performed at the discretion of the ECTU statistician. All imputations (if any) will be detailed in the statistical result report.

5.2 Quality control (QC) of summary tables and statistical analysis

Ahead of database lock and un-blinding, a full statistical report will be produced based on dummy randomisation codes, to allow for checking of the data and the proposed summaries/analyses.

Isolated data errors detected in the database as a result of the QC checks that are deemed significant will be submitted for enquiry to the trial manager or designee.

Systematic data errors in the data reporting will be investigated further; the data will be corrected if necessary, and the appropriate table then re-checked.

5.2.1 QC/Validation - Summary Tables

A random selection of unique analysis and summary tables will be QC'd using manual

methods (i.e. comparison of results in the table to results calculated by a calculator, spreadsheet, database output or any alternative summarisation tool).

5.2.2 QC/Validation - Statistical Analysis

QC/Validation of statistical analyses will be performed by peer review of program code, log and output. Additionally, the primary outcomes analysis will be replicated independently by a second statistician.

5.3 Un-blinding procedures

Not applicable

6 Population for analysis

6.1 Intention-to-treat population

The intention-to-treat (ITT) population will comprise all patients who have been randomised into the RESTART study, regardless of whether they were subsequently deemed ineligible after independent review of their diagnostic brain imaging.

6.2 Datasets for analysing the main study and the imaging sub-studies

The main report of the RCT will include the entire ITT population.

However, in the brain imaging studies within RESTART, imaging may: not have proceeded despite consent being obtained; my not have been provided; may have been undertaken but may have contravened the required protocol; may have been undertaken, but was degraded by motion artefact; have demonstrated that the patient was ineligible for inclusion in RESTART (which precluded collection of ratings by the RESTART Imaging Panel), Therefore, patients will be included in the CT imaging study and the MRI sub-study datasets if their pre-randomisation brain imaging was readable and confirmed their eligibility for RESTART.

7 List of analyses to be performed

7.1 Recruitment and retention

No formal statistical testing will be performed. A CONSORT flow diagram will be provided. The statistical report will tabulate the number of patients consented, randomised, treated, adherent, and completed follow-up overall and by treatment group. The number of patients discontinued early from the study will be summarised by reason for withdrawal and treatment.

7.2 Baseline data: demographics, baseline/clinical characteristics

No formal statistical testing will be performed. The following will be presented and summarised by treatment group and overall:

- the covariates used in the minimisation algorithm (qualifying ICH location; time since ICH symptom onset; antiplatelet drug(s) that patient's physician would use if allocated; age; predicted six month outcome)

- sex; ethnicity; diagnostic imaging; MRI sub-study imaging; indicated uncertainty about starting antiplatelet drugs; functional status; modified Rankin Scale score; co-morbidities; antithrombotic drugs taken before ICH; timing of key events – symptom onset to randomisation – symptom onset to earliest imaging study – earliest imaging study to randomisation – symptom onset to sub-study MRI (if applicable) – sub-study MRI to randomisation (if applicable)

7.3 Protocol deviations/violations

Any change, divergence, or departure from the trial design or procedures defined in the protocol or Good Clinical Practice are identified and recorded as a deviation (if it does not significantly affect a participant's rights, safety, or well-being, or trial outcomes), or a violation (if the deviation may potentially significantly impact the completeness, accuracy, and/or reliability of the trial data or that may significantly affect a patient's rights, safety, or well-being). All protocol deviations/violations will be listed, but no formal statistical testing will be performed.

7.4 Adherence to allocated treatment

No formal statistical testing will be performed. Adherence will be reported descriptively per treatment group at discharge and at each annual follow-up.

7.5 Co-interventions

Use of anticoagulant drugs will be reported descriptively per treatment group at discharge and at each annual follow-up. Because blood pressure (BP) control is the other main confounder of the frequency of the primary outcome, the use of BP-lowering drugs at discharge after randomisation and at each annual follow-up will be reported (along with a summary of the available BPs of participants by treatment group).

7.6 Analysis of outcomes

The trial will be analysed on an intention-to-treat basis, incorporating all patients who were randomised and for whom outcome data are available, with patients analysed according to their allocated intervention, irrespective of whether the patient's actual management complied with the allocated intervention.

The survival function per treatment group will be estimated using a Kaplan-Meier survival analysis of time to first outcome event during follow-up from randomisation. Follow-up will be censored at death (unrelated to an outcome event), last available follow-up, or voluntary withdrawal from the trial. We will compare the survival function in the two trial arms using a Cox proportional hazards regression model, adjusting for all the covariates included in the minimisation algorithm (Qualifying ICH location of lobar *versus* non-lobar; Time since ICH symptom onset of 0-6 days *versus* 7-30 days *versus* over 30 days; Antiplatelet drug(s) that the patient's clinician would start if allocated to aspirin alone *versus* any other regimen; Participant's age at randomisation of less than 70 years *versus* 70 years or older; Predicted probability of a good six month outcome of less than 0.15 *versus* 0.15 or greater), and presenting the result as an estimated adjusted hazard ratio with its corresponding 95% CI. We will also report the unadjusted estimate of the hazard ratio and its corresponding 95% CI, together with the result of the logrank test. The absolute difference in event rates at one, two, three and four years will be estimated from the Kaplan-Meier analysis.

The proportional hazards assumption will be assessed graphically, and if there is strong evidence of violation of the assumption the impact on the analysis will be assessed by comparing the results of the pre-specified analysis with the results obtained using the restricted survival time approach.[*]

7.7 Primary outcome

The primary outcome event is the first fatal or non-fatal radiographically- or pathologically-proven recurrent symptomatic ICH.

---

[*] Royston P, Parmar MK. The use of restricted mean survival time to estimate the treatment effect in randomized clinical trials when the proportional hazards assumption is in doubt. *Statistics in Medicine* 2011;30(19):2409-21

7.7.1 Sensitivity analyses

We will conduct two exploratory sensitivity analyses of our primary analysis, by adding the following secondary outcomes to the primary outcome in the following order, to account for the possibility that some fatalities and non-fatal neurological events without adequate investigation may be recurrent ICH:

- Fatal or non-fatal symptomatic stroke consistent with the clinical manifestations of ICH, but without radiographic or pathological confirmation (or with brain imaging performed too late to distinguish ICH from ischaemic stroke).
- Deaths without a clear cause and without further investigation.

7.8 Secondary outcomes

The two key secondary outcomes are a composite of all serious haemorrhagic events and a composite of all serious vaso-occlusive events (including revascularisation procedures). These composites are defined specifically as:

7.8.1 All serious haemorrhagic events

This composite outcome includes all fatal or non-fatal symptomatic events (that are 'serious' because of their usual need for hospitalisation and influence on outcome and antithrombotic treatment):

- Radiographically- or pathologically-proven recurrent symptomatic ICH (the primary outcome)
- Other forms of symptomatic spontaneous or traumatic intracranial haemorrhage
    - Radiographically- or pathologically-proven spontaneous or traumatic extradural haemorrhage, subdural haemorrhage, subarachnoid haemorrhage, or intraventricular haemorrhage (not accompanying spontaneous ICH).
- Extracranial haemorrhage at any site requiring transfusion / endoscopic treatment / surgery, or resulting in death

7.8.2 All serious vaso-occlusive events (including revascularisation procedures)

This composite outcome includes fatal or non-fatal symptomatic events (that are 'serious' because of their usual need for hospitalisation and influence on outcome and antithrombotic treatment) or revascularisation procedures (we will not include transient ischaemic attack or retinal artery occlusion):

- Fatal or non-fatal vaso-occlusive events
    - Ischaemic stroke, acute coronary syndrome (unstable angina or myocardial infarction), mesenteric ischaemia or peripheral arterial occlusion.
    - Symptomatic deep vein thrombosis, pulmonary embolism.
- Carotid, coronary, or peripheral arterial revascularisation procedures.

7.8.3 Composite of all serious haemorrhagic or vaso-occlusive events

A further secondary analysis will be performed on the composite event which combines the two composites defined above, i.e. the composite of any serious haemorrhagic or vaso-occlusive event.

For completeness, the corresponding analysis will also be performed for each composite outcome event proposed in the trial protocol.

The interpretation of the trial findings will respect this prespecified hierarchy of primary outcome, key secondary outcomes and other secondary outcomes, and no formal

adjustment will be made to significance levels to allow for multiplicity.

7.8.4 Sensitivity analysis

A sensitivity analysis will be performed to reflect the cumulative incidence of serious haemorrhagic or vaso-occlusive events. This analysis will use re-randomisation tests, as described by Ford *et al*.[†]

7.8.5 Annual assessment of modified Rankin Scale (mRS)

A separate analysis will be performed for each annual assessment. The analysis at year 'x' will be restricted to those recruits who were randomised at least 'x' years prior to study close, to avoid including early deaths in the relevant follow-up year when the corresponding surviving recruits would not have had the potential to be assessed. The analysis will comprise a tabulation of mRS by randomised group, with the formal analysis being based on a Mann-Whitney test. Participants' type of domicile will be described as categorised on the discharge form and each annual participant questionnaire.

7.9 Sub-group analyses

A priori sub-groups for the primary analysis will be explored as follows:

- The five covariates used in the minimisation algorithm
- Pre-ICH antithrombotic drug regimen (antiplatelet *versus* anticoagulant)

We will also perform sub-group analyses according to whether a history of atrial fibrillation was documented as a co-morbidity at randomisation.

These sub-group analyses will be applied to the primary outcome and the key secondary outcomes: (i) all serious haemorrhagic events, (ii) all serious vaso-occlusive events (including revascularisation procedures), and (iii) the composite of all serious haemorrhagic or vaso-occlusive events.

These analyses will be performed by including an interaction term between treatment group and the relevant covariate in the Cox proportional hazards regression model described in Section 7.6 above. For the subgroup analysis relating to time since ICH symptom onset, two subgroups will be defined based on the time from onset being above or below the median time observed in the trial.

7.10 Serious adverse events

Serious adverse events (SAEs) are reported in RESTART if they not outcome events or expected complications of stroke. SAEs will be grouped by body system and for each event and each grouped set of events the number of events and the number of individuals experiencing at least one such event will be tabulated per randomised group.

7.11 Imaging sub-studies

The analysis of the imaging sub-studies will be largely exploratory. The MRI sub-study will test hypotheses about microbleeds and explore hypotheses about other modifiers of the effect of antiplatelet drugs. The diagnostic CT imaging study will explore hypotheses about modifiers of the effect of antiplatelet drugs.

The RESTART Imaging Panel's report of MRI sub-study imaging will describe the baseline characteristics of the participants' brains (e.g. brain microbleed

---

[†] Ford I, Murray H, McCowan C, Packard CJ. Long-Term Safety and Efficacy of Lowering Low-Density Lipoprotein Cholesterol With Statin Therapy: 20-Year Follow-Up of West of Scotland Coronary Prevention Study. *Circulation* 2016;133(11):1073-80

presence/location and burden; old infarcts or haemorrhages; superficial siderosis; white matter hyperintensities; enlarged perivascular spaces; and atrophy) and ICH (e.g. location, intraventricular extension; subarachnoid extension; and ICH volume). In the MRI sub-study, the focus of our hypothesis will be on testing whether the presence, number, or location of brain microbleeds modifies the effect of antiplatelet drugs on the primary outcome, adjusted for the same covariates used in the primary analysis of the entire RCT (although time since ICH will be grouped into 0-30 versus >30 days in view of the distribution of outcomes in the whole trial dataset. Should any of these adjusted regression models fail to converge then the corresponding unadjusted analysis will be reported instead. [before database lock and un-blinding to treatment allocation]). For dichotomous analysis the number of brain microbleeds will be split as 0 or 1 versus 2 or more and for categorical analysis the split will be 0 or 1 versus 2 to 4 versus 5 or more. Brain microbleed location will be grouped as strictly lobar versus other.

The RESTART Imaging Panel's report of diagnostic imaging will describe the baseline characteristics of the trial population's brains (e.g. old vascular lesions, periventricular lucencies, and atrophy) and ICH (e.g. location, intraventricular extension, subarachnoid extension, ICH volume, and finger-like projections[‡]).

8 Data Sharing

A set of files, containing the final data will be prepared, along with their corresponding data dictionary. These will be made available to the Chief Investigator at the end of the analysis phase.

Ownership of the data arising from this study resides with the Trial Steering Committee. Access to the datasets generated and/or analysed during RESTART will be available on reasonable request after the publication of the main results. Access will be controlled by the chief investigator, with the approval of the Trial Steering Committee.

‡ Rodrigues MA, Samarasekera N, Lerpiniere C, Humphreys C, McCarron MO, White PM, Nicoll JAR, Sudlow CLM, Cordonnier C, Wardlaw JM, Smith C, Al-Shahi Salman R. The Edinburgh computed tomography and genetic diagnostic criteria for lobar intracerebral haemorrhage associated with cerebral amyloid angiopathy: model development, internal validation and diagnostic test accuracy study. *The Lancet Neurology* 2018;17:232-40

Appendix 1: Outcome measures specified in the protocol

Primary

- **Fatal or non-fatal radiographically- or pathologically-proven recurrent symptomatic ICH.**

Secondary

In addition to the primary outcome defined above these additional outcome events will be captured for individual analysis and/or the derivation of composite endpoints:-

- **Fatal (i.e. followed by death within 30 days) or non-fatal (i.e. not followed by death within 30 days) serious vascular events:**
    - Symptomatic haemorrhagic events
        - Symptomatic spontaneous or traumatic extradural haemorrhage, subdural haemorrhage, subarachnoid haemorrhage, or intraventricular haemorrhage (not accompanying spontaneous ICH)
        - Symptomatic major extracranial haemorrhage, sub-divided by site (requiring transfusion or endoscopic treatment or surgery, or resulting in death within 30 days)
    - Symptomatic vaso-occlusive events
        - transient ischaemic attack
        - ischaemic stroke
        - acute coronary syndrome
        - peripheral arterial occlusion
        - mesenteric ischaemia
        - retinal arterial occlusion
        - deep vein thrombosis
        - pulmonary embolism
        - revascularisation procedures (carotid, coronary, or peripheral arterial)
        - cardiac death with symptoms suggestive of myocardial ischaemia (type 3), or evidence of arrhythmia
    - Symptomatic stroke of uncertain sub-type
        - Non-fatal stroke, with brain imaging performed too late to distinguish ICH from cerebral infarction
        - Rapidly fatal stroke, but without radiographic or pathological confirmation
- **Other fatal events**
    - Deaths without a clear cause and without further investigation
    - Deaths from any other cause

Annual ratings of participant function completed by participant or their carer

- Simplified modified Rankin Scale postal questionnaire
- Structured telephone interview with non-responders to the postal questionnaire

Appendix 2: Statistical extracts from the protocol

Sample size calculation

There is considerable uncertainty about the absolute risks of recurrent symptomatic ICH among survivors who were taking antiplatelet drugs at the time their index event, but a review of the literature suggests that the event rate lies in the range of about 1.8 to 7.4% per annum. Similarly, information about the relative increase in the risk of recurrent ICH on antiplatelet drugs is scarce, but estimated relative risks from non-randomised studies have ranged from no excess (RR=1) to a 4-fold excess (RR=4). This pilot study of 720 patients will have excellent power (after all participants have been followed for at least two years) to detect a doubling of the rate of ICH if the true rate is 4.5% per annum, but there would be 93% power at the 5% significance level to detect a 4-fold increase in risk of recurrent ICH if the annual risk is only 1%. In both these scenarios the absolute excess risk of recurrent ICH may be higher than any plausible benefit of treatment, in which case it may be inappropriate to consider a larger trial designed to demonstrate net benefit. Previous meta-analyses of RCTs of antiplatelet drugs have provided reliable information on the relative effects of antiplatelet regimens in the sorts of people with vaso-occlusive disease who will have been recruited into RESTART, so information about the overall rates of serious vascular events (for both arms combined) will be used together with reasonable assumptions about the effects of antiplatelet drugs on such events in order to assess the plausibility of a net benefit emerging in a larger main study.

The TSC will review the target sample size and adjust this based on accruing data on: the number of primary outcome events, completeness of follow up, and the enrolment into specific pre-specified subgroups (e.g. lobar ICH location).

Contraindications to MRI, claustrophobia, non-attendances, and scheduling constraints lead to an attrition of ~25% of patients, so we would hope to obtain **brain MRI on ~550 patients** before randomisation. To maximise our chances of recruiting the target number of patients with brain MRI, we will target the set-up of sites where brain MRI is available in the first 3-6 months of the start-up phase of RESTART.

Proposed analyses

Our provisional analysis plan is described below, but we intend to publish a final Statistical Analysis Plan before the database is locked for analysis and the results are known. In order to preserve fully the huge benefit of randomisation, we will include all randomised participants in the analysis (irrespective of whether they adhere to the allocated treatment), all retained in the group to which they were allocated (i.e. “as-randomised”). This will comprise a Kaplan Meier survival analysis of time to first outcome event after randomisation. Follow-up will be censored at death (unrelated to an outcome event), last available follow-up, or voluntary withdrawal from the trial. We will compare the survival function in the two trial arms using a Cox proportional hazards regression model, adjusting for all the covariates included in the minimisation algorithm, and presenting the result as an estimated adjusted hazard ratio with its corresponding 95% CI. We will also report the unadjusted estimate of the hazard ratio and its corresponding 95% CI, together with the result of the logrank test.

Primary analysis

This will be restricted to the primary outcome of first fatal or non-fatal radiographically- or pathologically-proven recurrent symptomatic ICH.

Secondary analyses

Other possible manifestations of recurrent spontaneous ICH

We will conduct two exploratory sensitivity analyses of our primary analysis, by adding the following secondary outcomes to the primary outcome in the following order, to account for the possibility that some fatalities and non-fatal neurological events without adequate investigation may be recurrent ICH:

- Fatal or non-fatal symptomatic stroke consistent with the clinical manifestations of ICH, but without radiographic or pathological confirmation (or with brain imaging performed too late to distinguish ICH from ischaemic stroke).
- Deaths without a clear cause and without further investigation.

Other potential composite outcomes

Other forms of symptomatic spontaneous or traumatic intracranial haemorrhage

- Fatal or non-fatal radiographically- or pathologically-proven spontaneous or traumatic extradural haemorrhage, subdural haemorrhage, subarachnoid haemorrhage, or intraventricular haemorrhage (not accompanying spontaneous ICH).

Fatal or non-fatal extracranial haemorrhage

- At any site (but this will mainly be upper or lower gastrointestinal bleeding), requiring transfusion / endoscopic treatment / surgery, or resulting in death.

Fatal or non-fatal vaso-occlusive events

- Transient ischaemic attack, ischaemic stroke, unstable angina, myocardial infarction, peripheral arterial occlusion, or retinal arterial occlusion.
- Symptomatic deep vein thrombosis, pulmonary embolism.
- Carotid, coronary, or peripheral arterial revascularisation procedures.

Composite outcome of serious vascular events

- Non-fatal myocardial infarction.
- Non-fatal stroke.
- Death from a vascular cause (including sudden death, pulmonary embolism, haemorrhage, and death from an unknown cause).

Sub-group analyses

We will perform the following sub-group analyses of the primary outcome, and test for sub-group interactions if appropriate:

- Qualifying ICH location (lobar versus non-lobar, based on local investigator's interpretation of scan)
- Time since ICH symptom onset (0-6 days, 7-30 days, > 30 days)
- Antiplatelet drug(s) that the patient's physician would start if allocated (aspirin alone versus other antiplatelet regimen [including combination treatment])
- Participant age at randomisation (<70 years versus 70 years or older)
- Predicted six month outcome (predicted probability of good outcome <0.15 versus ≥0.15)
- Pre-ICH antithrombotic drug regimen (antiplatelet versus anticoagulant)

Brain MRI sub-study

Brain microbleeds on MRI appear to be a biomarker of bleeding-prone microangiopathies (such as arteriolosclerosis and cerebral amyloid angiopathy), they predict recurrent ICH, and lobar microbleeds also appeared to modify the effect of aspirin on recurrent ICH risk in one observational study. Furthermore, a collaborative meta-analysis of case-case comparisons has found a preponderance of brain microbleeds amongst patients who had an ICH whilst taking antiplatelet drugs compared to those who were not on any antithrombotics when they had an ICH. So we intend to perform a brain MRI sub-study in ~550 of the 720 participants to better understand these microangiopathies by exploring whether the presence, number, or location of brain microbleeds modifies the effect of antiplatelet drugs on the primary outcome."
